# Coping strategies for financial burdens in families with childhood pneumonia in Bangladesh

**DOI:** 10.1186/1471-2458-10-622

**Published:** 2010-10-19

**Authors:** Nadia I Alamgir, Aliya Naheed, Stephen P Luby

**Affiliations:** 1 James P Grant School of Public Health, BRAC University, 66 Mohakhali, Dhaka-1212, Bangladesh; 2 Programme on Infectious Diseases and Vaccine Sciences (PIDVS), Health Systems and Infectious Diseases Division (HSID), International Centre for Diarrhoeal Diseases Research, Bangladesh (ICDDR,B), GPO Box-128, Dhaka-1000, Bangladesh

## Abstract

**Background:**

This study aimed to determine the out-of pocket expenditure and coping strategies adopted by families of children admitted in a hospital in Bangladesh with pneumonia.

**Methods:**

Trained interviewers surveyed parents of 90 children and conducted in-depth interviews with six families below the age of 5 years who were admitted to the largest pediatric hospital in Bangladesh with a diagnosis of pneumonia. We estimated the total cost of illness associated with hospitalization and explored the coping strategies of the families.

**Results:**

The mean expenditure of the families for the illness episode was US$ 94 (±SD 52.5) with 75% having spent more than half of their total monthly expenditure on this hospitalization. Three fourths (68/90, 76%) of the families managed the expenditure by borrowing, mortgaging or selling assets; 64% had to borrow the full cost of hospitalization and 10% borrowed from the formal sector with a monthly interest rate of 5 to 30%. The burden was highest for the people from poor income strata. Families earning ≤US$ 59 per month were 10 times more likely than families earning ≥US$ 59 per month to borrow money (OR = 10.0, 95% CI: 2.8-38.8). To repay their debts, 22% of families reported that they would work extra hours and 50% planned to reduce spending on food and education for their children.

**Conclusions:**

Coping strategies adopted by the families to manage the out-of-pocket expenditure for children requiring hospitalization were catastrophic for the majority of the families. Efforts to prevent childhood pneumonia for example, by vaccination against the most common pathogens, by improving air quality and by improving childhood nutrition can provide a double advantage. They can prevent both disease and poverty.

## Background

Pneumonia is remarkably common in Bangladesh. In a systematic population based assessment in one low income neighborhood in Dhaka, Bangladesh, the incidence of pneumonia among children under five years old was 0.51 episodes per child per year [1]. Pneumonia is the leading cause of death among children in Bangladesh [2]. A substantial portion (43%) of deaths in infancy and 21% of deaths in children under five years old were associated with acute lower respiratory infection, especially pneumonia [3]. When a child develops pneumonia, doctors prescribe antimicrobials and in severe cases the child is hospitalized [4].

In Bangladesh, where 49.8% of the total population lives below the national poverty line [5] and families pay for most of the costs of clinical care [6], hospitalizing a child for pneumonia is a substantial expense for low income families.

We conducted a study to measure the cost of hospitalization for pneumonia in a large pediatric hospital in Dhaka, Bangladesh, to identify the coping strategies adopted by families to manage the finances for treating pneumonia in hospitals and to explore the impact of these strategies on the families' future economic prospects.

## Methods

### Study site

We conducted the study in the inpatient department of Dhaka Shishu Hospital. This is the largest pediatric hospital in Bangladesh. It is a semi-autonomous body. The Government of Bangladesh subsidizes 50% of the operating costs of this 450 bed hospital and the remaining expenditures are supported by patients' fees, donations, and grants. Fifty percent of the hospital beds are non-paying beds where the hospital attempts to pay all costs, including patient care, first line treatment and food [7]. The demand for the nonpaying beds greatly exceeds the supply, so even poor patients are usually first admitted to a paying bed, and then transferred to a nonpaying bed when one becomes available. In addition to bed costs since the supply of drugs and equipments were not adequate most patients had to bear the expenses for the use of drugs and equipments even in the non-paying beds.

### Study population, sampling and sample size

We enrolled 90 children aged 0-5 years in November and December 2007 who were admitted to Dhaka Shishu Hospital with a physician's diagnosis of pneumonia, severe pneumonia or very severe pneumonia noted on their treatment records. Patients with other complications in addition to pneumonia such as diarrhea, severe malnutrition or congenital heart disease or heart failure and patients admitted in the private cabins were excluded to reduce possible overestimation of the cost.

We enrolled patients from both paying and non-paying beds shortly after their admission. We classified patients based on what type of bed they occupied at the time of admission, even if they later switched to another type. We also estimated cost incurred by the families since the onset of illness, which included their health seeking behavior before coming to the hospital.

Our primary outcome was the proportion of households who borrowed money to treat their children. We assumed that it would be approximately 70% [6]. To estimate this proportion ± 10% at 95% confidence required that we enroll 81 patients. We enrolled 90 anticipating that up to 10% would drop out.

We purposively selected six families for in-depth interview, three from rural and three from urban areas. Our intention was to collect a wide variety of information on the coping behaviors of extremely poor households, and to explore potential differences between urban and rural households. During the structured interview if we found a poor family, having a real hardship to manage finances (either from rural or urban) we selected that family for in-depth interview.

### Variables

Interviewers collected information on various characteristics of the study participants. Demographic characteristics included age, education, marital status, and residence. Socio-economic characteristics included occupation and annual and monthly household expenditure. Household expenditure was considered as a proxy of household income. We looked at the distribution of monthly household expenditure in our sample and divided it into thirds to derive their income strata.

Interviewers collected information on their health seeking behavior, out-of-pocket expenses incurred before and during hospitalization. These costs presented here are for a single hospitalization. It included both direct medical and non-medical costs. Medical costs in the hospital consisted of consultation fees, admission fees, bed rent, diagnostic tests and drugs. Non-medical costs consisted of travel, food and tips.

To identify coping strategies we explored the source of funds, amount of money borrowed, any loan repayment strategy, length of repayment, interest charged for borrowed amount and the overall impact of this borrowing on their basic livelihood expenditure.

### Data collection tools and techniques

The study design incorporated both quantitative and qualitative components. Trained experienced interviewers used a semi-structured pre-tested questionnaire for the quantitative component and a checklist for the qualitative interview and conducted a face to face interview. Both the questionnaire and the check-list were translated into *Bengali*, the national language.

We interviewed families three times: initially shortly after admission, a second time during hospitalization and finally at the time of hospital discharge. During the first interview we collected demographic and socioeconomic information; for the second and third interview we focused on expenditures. We interviewed either of the parents or, in some cases, both parents.

### Data analysis

We used descriptive statistics for the demographic and socioeconomic characteristics of the study participants. To compare the proportion of categorical variables we used the chi square test or when the expected cell size was <5, Fisher's exact test. We report mean and median expenditures. Because these data were skewed we evaluated whether expenditures differed between groups using the Mann Whitney test.

We multiplied costs for each component with the appropriate number. For drugs, the unit cost was first multiplied by the dosage number in each day and then with the total hospital days. Different medical supplies such as saline sets, intravenous fluids and diagnostic costs were usually a one time cost. Travel costs, food costs, and bed rent were estimated according to the duration of the hospital stay.

We defined out of pocket payments as all payments made by the family when seeking health care. We calculated frequencies, percentages and distribution of all kinds of expenditures. To calculate the total cost per patient for the episode of pneumonia, we summed out-of-pocket payments made by the families before and after coming to the hospital. In case of families shifted from paying bed to non-paying bed we estimated the total expenditure incurred.

For the qualitative part of the study we transcribed the interviews verbatim initially and then translated them into English, applying open codes to data representing significant sections of text. We then grouped these into organizing categories or themes. To develop the coding frame the research team constantly modified and checked these categories. The coding frame was influenced by ideas arising during data collection.

### Ethical Considerations

Interviewers took informed verbal consent from each of the respondents (parents of the patient) before starting the interviews. The study was approved by the Bangladesh Rural Advancement Committee (BRAC) University Ethics Committee.

## Results

### Socioeconomic characteristics of participating families

The median monthly household expenditure was $69.97 (range $14.57-$379; mean $75) which was somewhat higher than the average national household income of the country which is around $37 per month [5]. Nine percent of the population was below national poverty line; who earned <US$ 1 per day per family [8]. We found 38% of our study participants were from poor income strata (monthly expenditure ≤ US$59 per month). Forty-five percent of the primary wage earners in the family were office workers, businessmen, school teachers and factory workers who had a fixed monthly income. The remaining 55% included agricultural workers, daily laborers, seasonal workers, and workers associated with other informal sectors (i.e., beggars, hawkers, street vendors and carpenters). Three percent of the family heads were unemployed. Forty-eight percent of the families resided in rural areas (Table 1).

**Table 1:** Out of pocket Expenditure by Socio demographic characteristics and bed status at Dhaka Shishu Hospital, December 2007

Out of Pocket expenditure				**P value**^**b**^
	**Income strata**^**a**^			

	N = 90	Poor (n = 34)	High (n = 30)	

Mean	US$94 (±SD52.5)	US$ 62	US $ 94	

Median	US$82	US$ 71	US $ 124	0.11

Range	US$(4-430)	US $ (18-201)	US $ (19-422)	

	Residence type			

		Rural (n = 43)	Urban (n = 47)	

Mean		US$ 65.43	US$ 55.56	

Median		US$ 50.44	US$ 40.80	0.35

Range		US$ (12-165)	US $ (10-112)	

	Bed status			

		Paying (n = 46)	Non-paying (n = 44)	

Mean		US$ 50	US$ 12	

Median		US$ 65	US$ 30	0.23

Range		US$ (5-120)	US$ (0-100)	

^a ^Income strata:. Poor households earned ≤US$59 per month, and high households >US$103 per month. We considered monthly expenditure is a proxy of monthly income.

^b ^For test of difference Mann Whitney test were done.

During the data collection period we identified 116 patients in the admission register with a diagnosis of pneumonia. According to the individual patient treatment sheet 26 of these patients had other complications in addition to pneumonia, and so we excluded them and enrolled 90 patients. The median age of the patients was five months (range 0-5 years); 67% were male. Forty-eight percent of the patients were diagnosed with pneumonia and 52% with severe pneumonia. Ten patients (11%) left the hospital without completing their treatment. During the study period four children (4.4%) died. The median duration of hospitalization was eight days (range 1-34 days). Eighty one percent of the patients were utilizing paying beds at the time of the initial interview. Eighty one percent of the patients were utilizing paying beds at the time of the initial interview. Nineteen (21%) patients were later shifted to non paying beds.

### Health seeking behavior

None of the families brought their child on the first day of the illness to the hospital. On average they waited for 7 days. Thirty-eight percent of families visited a qualified doctor at his/her private office, while 23% visited a hospital. The next most visited service provider was a pharmacy (22%). Seven percent sought health care from multiple health care providers. Beside the biomedical practitioners 10% of the families also sought care from traditional practitioners. They spent a median of $30 ($5-$35) prior to coming to Dhaka Shishu Hospital.

### Expenditure for current episode of illness

The median total out of pocket expenditure for patients interviewed were US$110 Families whose child was admitted initially to a paying bed reported higher out-of-pocket expenses, but with this small sample size these differences may have been due to chance (Table 1). The mean non medical cost per patient was US$36 (range1.5-84; median US$56) and of the mean medical costs were US$75 (range 2.7-96; median US$17). Medical costs represented approximately 71% of the total in hospital expenditure.

Overall, 75% (n = 83) of families spent more than 50% of their monthly expenditure for the current illness episode (Table 2). Among the 34 lower income families (monthly expenditure ≤ 59) 28 (82%) spent more to treat the current illness than they earn in one month. Thirty percent of patient originally admitted to non-paying beds and 60% patient from the paying bed spent more than 50% of their income for this treatment purpose.

**Table 2:** In hospital out of pocket payment as percentage of total household income by income group

Income strata	No. of households	% of income spent for hospital cost among households, n (%)		
		
		≤ 50%	51-100%	**≥100%**^**d**^
Poor^a^	34	1 (3)	5 (15)	28 (82)

Middle^b^	26	1(4)	9 (35)	14 (54)

High^c^	30	13 (43)	15(50)	2 (6)

Total	90	15 (17)	31 (34)	44 (49)

^a^Monthly expenditure (≤US$59).

^b^Monthly expenditure (US$59 to US$103).

^c^Monthly expenditure (≥US$103).

^d^(≥100% indicates % of income spent for hospital cost was more than their monthly expenditure).

### Coping strategies for dealing with this financial burden

Families reported their major coping mechanism as spending household savings, borrowing money and or selling or mortgaging assets. Only 16% of the families could manage entirely with the money from their regular family income. Sixty-one percent of families were exclusively dependent on external financial sources. They managed finances from the informal sector such as a contribution/loan from relatives, friends or their employer (50%) and borrowing from the local moneylenders (11%), micro-finance schemes set up by nongovernmental organizations, or banks. Ten families (11%) mortgaged or sold household assets (jewelry, furniture or cattle) or land (Table 3).

**Table 3:** Borrowing money and borrowing strategy by SES and bed type

Borrowing money		Bed type		Income Strata	
	N = 90	Paying (n = 27)^a^	Non-Paying (n = 11)	Poor (n = 22)	High (n = 7)

Mean	US$57(SD ± 44)	US$90	US$30	US$ 47	US$ 12.

Median	US$55	US$105	US$60	US$ 53	US$ 30

Range	US$(0-255)	US$(2-30)	US$(0-70)	US$(19-154)	US$(0-19)

**Borrowing strategy**				N = 22	N = 7

Contribution/loan from relatives/friends	27(30%)	19 (70%)	8 (29%)	16 (72%)	6 (85%)

Local moneylenders	11(12%)	7 (70%)	4 (40%)	10 (45%)	1 (14%)

Mortgaged or sold household assets	10(11%)	8 (80%)	2 (20%)	6 (27%)	0

^**a **^n indicates the number of families borrowed money or adopted a strategy

The median amount borrowed per household was US$55 and was equivalent to 10% (range 0.58%-108%) of the monthly household expenditure. Rural households borrowed almost double the proportion of their household expenditures (median 14%; range 2%-108%) compared with urban households (median 6%; range 0.58%-28%).

Families who borrowed money were ten times more likely to be low income, compared to people who did not borrow money OR = 10.0 (95% CI: 2.8-38.8). Wealthy families were more likely to use family savings to meet the costs.

### Loan repayment strategy, time and interest rate

**Figure 1:**
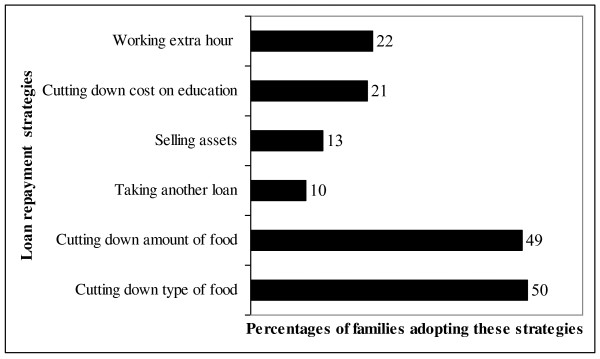
Different types of loan repayment strategy families planned to adopt.

Half of the respondents (50%) planned to reduce the type and amount of food their family purchased to repay their loan (Figure 1). Twenty-two percent were planning to repay the loan by working extra hours, while (13%) of them said that they would have to sell their assets to repay the loan. Twenty-two percent of families planned to stop their children's education or private tuition and/or shift their child to a lower cost school.

Most often (33%) money was borrowed from friends and relatives without interest. When borrowing was from moneylenders (n = 10), households reported interest rates of 5%-30% per month. Three households put up jewelry, land and household goods as security when borrowing money from moneylenders. Forty-six percent of families that borrowed money stated that they would need more than one year to repay the loan.

*"*Ayesha Akhter was eating her first meal of rice in over three days. *She sighed, 'If I can't be alive how will l be able to keep my baby alive?' Ayesha was the mother of a one year old baby. 'My husband left me. I beg for my livelihood. I borrowed from an NGO with high interest rate, but have no idea how I am going to repay my loan."*

### Impact of loan on the families

The families expressed many concerns about the unanticipated financial burden including wage loss, borrowing money, selling or mortgaging assets and finding the additional money needed to complete medical treatment. Fifteen percent of respondents, who planned to work hard to repay their loan, assumed that due to overwork their health status might deteriorate. Ten of the families who planned to change their dietary intake were also concerned that due to less intake of food their nutritional status might suffer. Respondents expressed anxiety about forfeiting their mortgaged assets.

"Samina had multiple episodes of pneumonia. The family was from the northern part of the country, where people have recently experienced seasonal food insecurity. This can force the poorest families to sell their assets for survival. While Samina's family was leaving the hospital against the advice of the physicians, her father sighed and whispered, *'It is Allah who determines birth and death. We sold our family assets to manage the money for the treatment of the child. If we stay here for one more day, we will have to live without food."*

Sixty-three percent of families who borrowed money from different sources responded that their personal relationships with family and friends would deteriorate if they could not repay the loan on time. Many thought that they would be verbally abused and harassed by the moneylender.

Rahela had five sisters and only one brother. She explained, "*Our brother was dying. We did not have any money even to bring him to the hospital. I borrowed money from an uncle in the neighbourhood. I don't know how we will be able to repay the loan. Due to the expenses incurred for this treatment, we will have to stop treatment of one of our sisters. She is disabled. She needs regular medication. Our father may need to work in other peoples' land now. We may also have to stop our education. Moreover, if we can't repay the loan timely, the marriages of my sisters will also be at stake.*"

"Kulsum had to mortgage her jewellery to a local money lender in order to arrange money for the treatment of her baby. She said, *'I mortgaged my jewellery to borrow the money. If I don't pay the interest regularly, the amount will increase manifold with compound interest and I will not be able to release the jewellery.'*

"Prodip Mondol, a fisherman from rural area, who lives on a seasonal income, explained why they were planning to take early discharge on request from the hospital.

'I don't have any regular income. I have a very big family. We live from hand to mouth. Every month I need to borrow money even to buy food. This is 'monda' time (bad time) for me. I borrowed money with interest. But the money is already exhausted and I don't know how long it will take to repay the loan."

## Discussion

Childhood pneumonia is a minor cause of mortality in high income countries, but the leading cause of death of children in low income countries [9]. Pneumonia deaths can be prevented by using effective vaccines improving nutrition and air quality, and by prompt and appropriate treatment of respiratory illness in children[10]. Hospitalization for pneumonia can be critical for child survival, but this study illustrates that the financial cost can overwhelm family finances. Sixty-six percent of all participating families, and 82% of the families from the lowest income strata in our study, spent more than 50% of their monthly expenditure to cover the cost of hospitalization for pneumonia. For low income families this level of expenditure often forces households to cut their consumption of essential items, trigger productive asset sales or high levels of debt, and leads to impoverishment [11]. This downward spiral of loss of productive assets, and reduced income for food and children's education forces many families to slide into abject poverty and perpetuate the cycle of poverty to the next generation [12]. In Bangladesh, where half the total population lives below the national poverty line [5], these out-of-pocket health care costs increase the number of ultra poor.

These high costs also deter families from seeking clinical care for pneumonia [12,13]. In the 2007 Bangladesh Demographic and Health Survey children living in the poorest 20% of households in Bangladesh were 75% less likely to seek care for symptoms of an acute respiratory illness at a health facility or by a medically trained provider, compared to children living in the richest 20% of households [2].

Some researchers have proposed that careful use of user fees could subsidize care for the poor and actually reduce inequities [14]; however, cross subsidies that would lower the cost of care for poor families are typically unavailable in Bangladesh. Most of the cost of health care in Bangladesh is paid by families [15]. Even access to government health care which is purportedly free of charge actually requires payment of numerous unofficial fees [15]. Low income families are at high risk of insufficient care. In low income countries a typical household spends 2-5% of their income on health care [16]. However, in this study regardless of income groups, the healthcare expenditure was in all cases more than 10% of per capita income. When clinical care is dependent on the ability to pay, children living in the poorest families face the worst consequences [17].

These findings are consistent with several other studies from developing low income countries that show poorer families generally lack access to formal health insurance, credit and savings arrangements. Thus, much of the saving and borrowing by these households is informal and relies on the social capital of communities, such as borrowing from friends or relatives [18,19]. Research in Africa, rural China, Thailand and Vietnam concluded that future welfare can be at risk by incurring debts, selling off productive assets, or sacrificing investment in future productivity, for example by curtailing children's education and so triggering a vicious cycle of impoverishment and more indebtedness [20,21].

An important limitation of this study is that it focused on only 90 cases from a single facility, so these findings are not representative of all of Bangladesh. It is also possible that different causes of pneumonia could have different costs, and so the short two-month enrollment of study subjects may not be representative of all causes of pneumonia. However, the study was conducted in the largest pediatric hospital in Dhaka, where nearly half of the patients came from outside the city. This was usual type of patient for this facility and pneumonia admissions occurs year-round [22]. Moreover, the principal findings of substantial costs of hospitalization and coping strategies that threaten impoverishment have been noted with other health conditions in Bangladesh [19] and with pneumonia in other countries [9], so it is unlikely that these findings are exceptional.

A second limitation is that the study did not measure the direct expenditures for the follow-up costs after the child was discharged. In addition the study did not measure indirect costs, including wage loss from bringing the child to the hospital or future lost earnings from worsening child nutrition and education which may hamper their future well being. Thus, the reported costs should be interpreted as minimum costs.

## Conclusions

This study illustrates that hospitalization for pneumonia can easily become a catastrophic expenditure for the poor. Thus, efforts to prevent pneumonia are important both for child survival and to prevent worsening poverty. Efforts to improve income among the poor, reduce childhood malnutrition, improve air quality and vaccination against leading causes of childhood pneumonia including HIB and *pneumococcus *can provide a double advantage. They can prevent both disease and poverty.

## Competing interests

The authors declare that they have no competing interests.

## Authors' contributions

NIA, AN and SPL designed the study. NIA extracted the data, performed the statistical analysis and undertaken the first draft of manuscript. SPL and AN revised the manuscript for important intellectual content by providing comments and changes. SPL has coordinated the project. All authors read and approved the final manuscript.

## Pre-publication history

The pre-publication history for this paper can be accessed here:

http://www.biomedcentral.com/1471-2458/10/622/prepub
